# Antimicrobial resistance in patients with COVID-19: a systematic review and meta-analysis

**DOI:** 10.1016/S2666-5247(22)00355-X

**Published:** 2023-03

**Authors:** Bradley J Langford, Miranda So, Marina Simeonova, Valerie Leung, Jennifer Lo, Tiffany Kan, Sumit Raybardhan, Mia E Sapin, Kwadwo Mponponsuo, Ashley Farrell, Elizabeth Leung, Jean-Paul R Soucy, Alessandro Cassini, Derek MacFadden, Nick Daneman, Silvia Bertagnolio

**Affiliations:** a Public Health Ontario, Toronto, ON, Canada; b Dalla Lana School of Public Health, University of Toronto, Toronto, ON, Canada; c Leslie Dan Faculty of Pharmacy, University of Toronto, Toronto, ON, Canada; d University Health Network, Toronto, ON, Canada; e Sunnybrook Health Sciences Centre, Toronto, ON, Canada; f Toronto East Health Network, Toronto, ON, Canada; g North York General Hospital, Toronto, ON, Canada; h School of Epidemiology and Public Health, University of Ottawa, Ottawa, ON, Canada; i University of Calgary, Calgary, AB, Canada; j Alberta Health Services, Calgary, AB, Canada; k Unity Health Toronto, Toronto, ON, UK; l Antimicrobial Resistance Division, WHO, Geneva, Switzerland; m Ottawa Hospital, Ottawa, ON, Canada

## Abstract

**Background:**

Frequent use of antibiotics in patients with COVID-19 threatens to exacerbate antimicrobial resistance. We aimed to establish the prevalence and predictors of bacterial infections and antimicrobial resistance in patients with COVID-19.

**Methods:**

We did a systematic review and meta-analysis of studies of bacterial co-infections (identified within ≤48 h of presentation) and secondary infections (>48 h after presentation) in outpatients or hospitalised patients with COVID-19. We searched the WHO COVID-19 Research Database to identify cohort studies, case series, case-control trials, and randomised controlled trials with populations of at least 50 patients published in any language between Jan 1, 2019, and Dec 1, 2021. Reviews, editorials, letters, pre-prints, and conference proceedings were excluded, as were studies in which bacterial infection was not microbiologically confirmed (or confirmed via nasopharyngeal swab only). We screened titles and abstracts of papers identified by our search, and then assessed the full text of potentially relevant articles. We reported the pooled prevalence of bacterial infections and antimicrobial resistance by doing a random-effects meta-analysis and meta-regression. Our primary outcomes were the prevalence of bacterial co-infection and secondary infection, and the prevalence of antibiotic-resistant pathogens among patients with laboratory-confirmed COVID-19 and bacterial infections. The study protocol was registered with PROSPERO (CRD42021297344).

**Findings:**

We included 148 studies of 362  976 patients, which were done between December, 2019, and May, 2021. The prevalence of bacterial co-infection was 5·3% (95% CI 3·8–7·4), whereas the prevalence of secondary bacterial infection was 18·4% (14·0–23·7). 42 (28%) studies included comprehensive data for the prevalence of antimicrobial resistance among bacterial infections. Among people with bacterial infections, the proportion of infections that were resistant to antimicrobials was 60·8% (95% CI 38·6–79·3), and the proportion of isolates that were resistant was 37·5% (26·9–49·5). Heterogeneity in the reported prevalence of antimicrobial resistance in organisms was substantial (*I*^2^=95%).

**Interpretation:**

Although infrequently assessed, antimicrobial resistance is highly prevalent in patients with COVID-19 and bacterial infections. Future research and surveillance assessing the effect of COVID-19 on antimicrobial resistance at the patient and population level are urgently needed.

**Funding:**

WHO.

## Introduction

Frequent use of antibiotics in patients with COVID-19 has the potential to worsen antimicrobial resistance. A systematic analysis^1^ published in 2022 suggested that 0·9–1·7 million deaths were attributable to bacterial antimicrobial resistance in 2019, which would make resistance one of the leading causes of mortality globally. Antibiotics are often empirically prescribed for suspected bacterial infections both at hospital admission and during hospital stays, which increases the risk of resistance. Published reports^2^, ^3^, ^4^, ^5^, ^6^, ^7^ suggest that the prevalence of bacterial co-infections (ie, infection diagnosed within the first 48 h of hospital admission) among patients with COVID-19 is generally low (3–8%) but antibiotic use is high (50–75%), although variable. Emerging evidence suggests that surges in COVID-19-related hospital admissions are associated with an increase in antibiotic-resistant infections, including methicillin-resistant *Staphylococcus aureus* and vancomycin-resistant *Enterococcus*.^8^, ^9^

Improved understanding of the prevalence of bacterial infections—and particularly of antibiotic-resistant bacterial infections—among patients diagnosed with COVID-19 would inform appropriate clinical management. We aimed to establish the prevalence and predictors of bacterial infections and antimicrobial resistance in patients with COVID-19 by assessing the available evidence.

## Methods

### Search strategy and selection criteria

We did a systematic review and meta-analysis guided by the Cochrane Rapid Reviews Methods Group^10^ to estimate the prevalence and predictors of bacterial infections and antimicrobial resistance among patients with a laboratory diagnosis of COVID-19. We included studies of hospitalised patients or a mix of hospitalised and non-hospitalised patients, and excluded studies that exclusively included patients in the community.

**Research in context**

**Evidence before this study**

The potential exacerbation of antimicrobial resistance as a result of inappropriate antibiotic use in patients with COVID-19 has been a major concern since the beginning of the COVID-19 pandemic. Published reports of patients with COVID-19 describe a low prevalence of bacterial co-infection (≤48 h after presentation), but a higher prevalence in critically ill patients, particularly as secondary bacterial infection (>48 h after presentation). Despite a low overall prevalence of bacterial infection (3–8%), antibiotic use is high (50–75%), prompting concerns about antibiotic overuse and selection for antimicrobial-resistant pathogens.

**Added value of this study**

Our systematic review and meta-regression analysis of data for 362 976 patients from 148 studies in more than 40 countries is, to our knowledge, the largest to focus on antimicrobial resistance associated with COVID-19. Our findings suggest that the likelihood of bacterial co-infections in patients presenting to hospital with COVID-19 is low. By contrast, in patients admitted to intensive care units, the prevalence of secondary infections is high. We estimated that the prevalence of antimicrobial-resistant bacterial infections among all hospitalised patients with COVID-19 and bacterial infections was 60·8% (95% CI 38·6–79·3; data from 17 studies), and 37·5% (26·9–49·5; data from 42 studies) of isolates were resistant. Study-level predictors for increased prevalence of antimicrobial resistance included low-income or middle-income setting, being in an intensive care unit, interleukin-6 inhibitor use, and diabetes.

**Implications of all the available evidence**

The prevalence of bacterial co-infections in patients presenting to hospital with COVID-19 is low. In contrast, in critically ill patients, the prevalence of secondary infections is high. A substantial proportion of bacterial infections in patients hospitalised with COVID-19 involve antimicrobial-resistant species, particularly among patients in intensive care units. Our findings highlight the need for global surveillance of antimicrobial resistance, and the importance of careful risk–benefit assessments of empirical antibiotic use in patients with COVID-19. With the global COVID-19 pandemic continuing, judicious use of antibiotics in affected populations is a crucial aspect of clinical practice around the world.

We did a comprehensive search of the WHO COVID-19 Research Database for cohort studies, case series, case-control trials, and randomised controlled trials with populations of at least 50 patients in whom bacterial infections were investigated that were published in any language between Jan 1, 2019, and Dec 1, 2021. Reviews, editorials, letters, pre-prints, and conference proceedings were excluded. We did not search trial registries or seek data from unpublished studies. The WHO COVID-19 Research Database is an open-access, comprehensive source of COVID-19 literature that is updated weekly and includes citations from MEDLINE, Scopus, CINAHL, ProQuest Central, Embase, and Global Index Medicus.^11^ The search strategy was structured to include terms pertaining to bacterial infections, secondary infections, and antimicrobial resistance. The appendix (pp 2–3) contains a full list of the search terms used. Search results were imported into Covidence, an online tool for the management of systematic review data.

To ensure a reliable diagnosis of bacterial infections, we excluded studies in which infection was tested for using only nasopharyngeal swabs, and those in which infections caused by bacteria were not differentiated from those caused by fungi and viruses other than COVID-19. We also excluded studies in which bacterial infections were not microbiologically confirmed (ie, studies in which infections were diagnosed on the basis of biomarkers, leukocytosis, or serology). Bacterial infection was classified as either co-infection, secondary infection, or unspecified. We classified data as being related to co-infection if the study included the term “co-infection” or specified that an infection was diagnosed within the first 48 h of initial assessment or admission. We classified data as related to secondary infection if studies used the terms “secondary infection”, “super-infection”, or “healthcare-associated infection”, or stated that infection was identified >48 h after initial assessment.

Title and abstract screening was done by BJL, VL, MSo, MSi, ND, DM, SR, MES, and EL, all of whom independently selected studies on the basis of the inclusion and exclusion criteria. If articles seemed relevant, then the full text was assessed for inclusion. Because of the high volume of citations, and the substantial κ agreement (0·62–0·68) among members of the project team in previous COVID-19-related rapid reviews (unpublished), screening at both stages was done by one author only. The study protocol was registered under PROSPERO, the international registry of systematic reviews (CRD42021297344).

### Data analysis

Our primary outcomes were the prevalence of bacterial co-infection and secondary infection, and the prevalence and predictors of antibiotic-resistant pathogens among patients with laboratory-confirmed COVID-19 and bacterial co-infection or secondary infection. Pre-planned secondary analyses stratified data by health-care setting and type of bacterial infection (ie, co-infection *vs* secondary infection). A full list of secondary outcomes is provided in the trial protocol.

Data were extracted as reported study-level summary estimates. Initial data extraction was done by VL, BJL, MSi, JL, TK, and EL. For quality assurance, data collected from all the included studies were validated by a second team member (VL, BJL, TK, JL, MSi, EL, MSo, or MES) for accuracy and completeness. All discrepancies were reviewed and resolved either by consensus or by a third team member if consensus was not reached.

First, we identified the study setting (eg, all hospitalised patients irrespective of critical care status, critically ill patients only, a mix of outpatients and inpatients), the WHO geographical region in which the study was done,^12^ and study period. We extracted demographic data for age, sex, and the presence of comorbidities, as well as data about the drug class of any COVID-19 therapeutics used (eg, interleukin-6 [IL-6] inhibitors, corticosteroids). Antimicrobial resistance data were extracted at the study level as the proportion of patients with infections caused by any resistant bacteria of interest on the WHO global priority list of antibiotic-resistant bacteria (panel).^13^ Bacteria were classed as multidrug resistant if they were not susceptible to at least one drug across three or more antibiotic classes (or as defined by study authors).

Panel **Study definition of antibiotic-resistant organisms**

***Staphylococcus aureus***

Methicillin-resistant *S aureus*

Vancomycin-intermediate-susceptibility *S aureus*

***Enterococcus faecium* or *Enterococcus faecalis***

Vancomycin-resistant *Enterococcus*

***Streptococcus pneumoniae***

Penicillin-resistant *S pneumoniae*

Fluoroquinolone-resistant *S pneumoniae*

***Pseudomonas* species**

Carbapenem-resistant spp

Colistin-resistant spp

Multidrug-resistant* spp

***Acinetobacter baumannii***

Carbapenem-resistant *A baumannii*

Colistin-resistant *A baumannii*

Multidrug-resistant* *A baumannii*

***Stenotrophomonas* species**

Multidrug-resistant* spp

**Enterobacterales (eg, *Escherichia coli, Klebsiella* species, *Enterobacter* species, *Proteus* species)**

Enterobacterales resistant to third-generation and higher cephalosporins (eg, ceftriaxone, cefotaxime, ceftazidime, cefepime) or that produce extended-spectrum β-lactamase

Carbapenem-resistant Enterobacterales

Colistin-resistant Enterobacterales

Multidrug-resistant* Enterobacterales

***Haemophilus influenzae***

Ampicillin-resistant and amoxicillin-resistant *H influenzae*

Definitions are based on the WHO global priority list of antibiotic-resistant bacteria.

To estimate pathogen-specific resistance for each organism, we analysed any study that reported resistance or susceptibility for each specific organism. To minimise the risk of underestimation in our overall analysis of the prevalence of antimicrobial resistance, we included only studies in which susceptibility or resistance data were reported for four or more species (or for all reported species if fewer than four species were isolated). We chose a threshold of four species because our preliminary literature review found that reporting of antibiotic resistance is often restricted to few drug–organism combinations. We estimated the prevalence of bacterial infections stratified by setting, co-infection versus secondary infections, geographical area, and country income level.

We calculated the prevalence of antimicrobial resistance per patient (ie, the proportion of patients infected with resistant bacteria among the total number of patients with bacterial infections) and per organism (ie, number of resistant species out of the number of isolates identified). For assessment of species-specific antimicrobial resistance, the numerator was the number of isolates with a specific resistance mechanism (eg, extended-spectrum β-lactamase-producing *Escherichia coli*) and the denominator was the total number of organisms isolated in which susceptibility or resistance to a specific antibiotic was reported (eg, all *E coli* isolates from studies in which susceptibility or resistance was reported).

We used a validated ten-item risk of bias tool for disease prevalence. We classified the risk of bias as low (score ≥8), moderate (5–7), or high (0–4).^14^

We used random-effects meta-analysis (generalised linear mixed models) to pool the prevalence of bacterial infections and antimicrobial resistance across studies. Specifically, we used random intercept logistic regression with a logit link and estimated between-study variance using the maximum-likelihood estimator.^15^, ^16^ Heterogeneity was assessed with the *I*^2^ statistic.^10^ We also did a sensitivity analysis, stratified by study quality, to further estimate the robustness of the relevance estimates.

To understand the effect of specific population-level variables on bacterial infection and antimicrobial resistance, we did univariable meta-regression of patient characteristics, health-care settings, geographical region, and end month of study to assess temporal trends. Differences in the prevalence of bacterial infection and antimicrobial resistance for each variable were reported as the odds ratio (OR) compared with reference categories for every 10% increase in the proportion of patients with comorbidities and of those receiving COVID-19 therapeutics and for every 10-year increase in age. Multivariable meta-regression was then done to account for key study-level factors mediating the risk of infection, including mean and median patient age^17^ and severity of COVID-19 (an index including the highest value among the proportion of patients with acute respiratory distress syndrome, the proportion of patients in intensive care units [ICUs], and the proportion of patients who underwent mechanical ventilation).^18^ For each variable, we fit one unadjusted (univariable) model and one adjusted (multivariable) model. Only studies in which both the variables of interest and the adjustment variables were reported were used in model fitting (ie, the same subset of studies was used for the unadjusted and adjusted models). All analyses were done with the packages metafor and meta in R (version 4.1.2).

### Role of the funding source

Staff employed by the funder of the study had roles in study design, data interpretation, and writing of the report, but not in data collection or data analysis.

## Results

**Figure 1:**
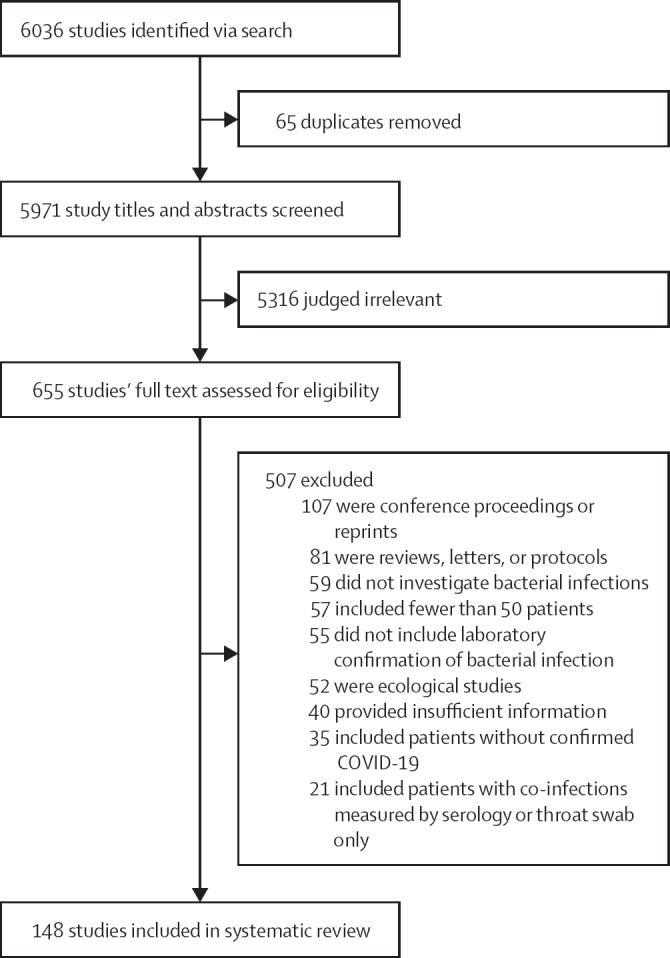
PRISMA flow diagram

6036 studies were identified via database searches, of which 148 (2%) were included in the analysis (figure 1). The 148 included studies were published between December, 2019, and May, 2021.^19^, ^20^, ^21^, ^22^, ^23^, ^24^, ^25^, ^26^, ^27^, ^28^, ^29^, ^30^, ^31^, ^32^, ^33^, ^34^, ^35^, ^36^, ^37^, ^38^, ^39^, ^40^, ^41^, ^42^, ^43^, ^44^, ^45^, ^46^, ^47^, ^48^, ^49^, ^50^, ^51^, ^52^, ^53^, ^54^, ^55^, ^56^, ^57^, ^58^, ^59^, ^60^, ^61^, ^62^, ^63^, ^64^, ^65^, ^66^, ^67^, ^68^, ^69^, ^70^, ^71^, ^72^, ^73^, ^74^, ^75^, ^76^, ^77^, ^78^, ^79^, ^80^, ^81^, ^82^, ^83^, ^84^, ^85^, ^86^, ^87^, ^88^, ^89^, ^90^, ^91^, ^92^, ^93^, ^94^, ^95^, ^96^, ^97^, ^98^, ^99^, ^100^, ^101^, ^102^, ^103^, ^104^, ^105^, ^106^, ^107^, ^108^, ^109^, ^110^, ^111^, ^112^, ^113^, ^114^, ^115^, ^116^, ^117^, ^118^, ^119^, ^120^, ^121^, ^122^, ^123^, ^124^, ^125^, ^126^, ^127^, ^128^, ^129^, ^130^, ^131^, ^132^, ^133^, ^134^, ^135^, ^136^, ^137^, ^138^, ^139^, ^140^, ^141^, ^142^, ^143^, ^144^, ^145^, ^146^, ^147^, ^148^, ^149^, ^150^, ^151^, ^152^, ^153^, ^154^, ^155^, ^156^, ^157^, ^158^, ^159^, ^160^, ^161^, ^162^, ^163^, ^164^, ^165^, ^166^ All included studies were observational, and most (n=120 [81%]) were retrospective cohort studies.

In these 148 studies, 362 976 patients were assessed for bacterial infections. 69 (47%) studies (n=253 777 patients) were done in Europe, 40 (27%; n=47 111) in the Americas, 15 (10%; n=4515) in the Western Pacific, 13 (9%; n=34 012) in the Eastern Mediterranean, eight (5%; n=22 287) in South-East Asia, and two (1%; n=684) in Africa. One study (n=590) was done in multiple regions. According to the World Bank classification,^167^ 112 studies (76%; n=330 745) were done in high-income countries, 35 (24%; n=31 641) in low-income and middle-income countries, and one in multiple regions.

87 (59%) studies included all hospitalised patients (n=119 257), 55 (37%) included patients in ICUs only (n=15 417), and six (4%) included both inpatients and outpatients (n=228 302; appendix pp 4–10). The number of patients in the ICU was reported in 120 (81%) studies, and the median proportion of patients in the ICU was 50% (IQR 20–100). The number of patients receiving mechanical ventilation was reported in 90 (61%) studies, and the median proportion was 46% (16–100).

Median or mean patient age and sex was reported in 127 (86%) studies. The median age was 62 years (IQR 59–65), and the median proportion of participants who were female was 35% (28–44). Two studies assessed neonatal or paediatric populations. Current or former smoking status was detailed in 28 studies (median proportion of population 17% [IQR 5–26]), chronic obstructive pulmonary disease in 65 studies (7% [4–11]), cardiovascular disease in 67 studies (15% [7–21]), diabetes in 93 studies (27% [20–37]), malignancy in 59 studies (7% [4–11]), and immunocompromising conditions in 63 studies (8% [5–12]).

Use of corticosteroids was described in 72 studies (used in a median of 36% of patients [IQR 22–62]), and use of IL-6 inhibitors was described in 56 (10% [4–41]). Use of antibiotics was described in 86 studies (75% [57–90]). Most studies had a low (n=81) or moderate (n=61) risk of bias. Six had a high risk of bias.

In 113 (76%) studies, microbiological culture was used to detect bacterial infections, and in 19 (13%) a combination of culture and urinary antigen testing was used (eg, for *Streptococcus pneumoniae* or *Legionella pneumophila*). In 14 (9%) studies, a combination of tests, including culturing, urinary antigens, and molecular techniques (eg, nucleic acid amplification or sequencing), were used, and two (1%) relied on nucleic acid amplification testing alone. In 108 (73%) studies, pre-specified clinical criteria were used to differentiate infection from colonisation or contamination. Samples (eg, blood, respiratory samples, urine) were collected from several anatomical sites in 96 (65%) studies. Respiratory specimens alone were collected in 29 (20%) studies and blood specimens alone in 22 (15%). The type of sample collected was not specified in one (1%) study.

**Figure 2:**
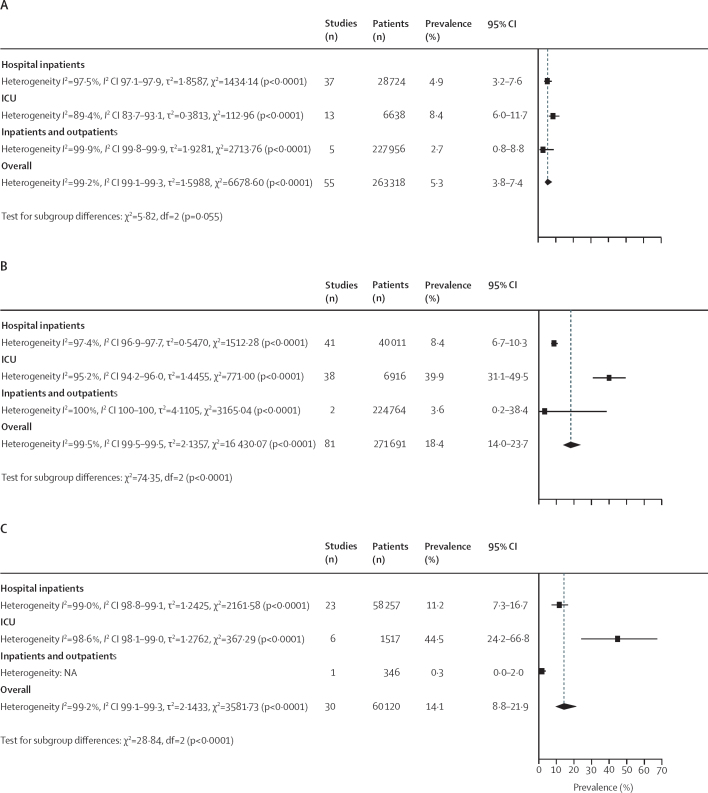
Forest plots of prevalence of bacterial co-infection in patients with COVID-19 (A) Bacterial co-infection. (B) Bacterial secondary infection. (C) Unspecified bacterial infections. ICU=intensive care unit. NA=not applicable.

The prevalence of bacterial co-infection or secondary infection, or both, was reported in 138 (93%) studies (n=355 579 patients). When we pooled data across all eligible studies, the prevalence of bacterial co-infections was 5·3% (95% CI 3·8–7·4), and the prevalence of secondary infections was 18·4% (14·0–23·7; figure 2). Co-infection was reported in 4·9% (3·2–7·6) of hospitalised patients and 8·4% (6·0–11·7) of ICU patients (figure 2). Secondary bacterial infection was identified in 8·4% (6·7–10·3) of hospitalised patients and 39·9% (31·1–49·5) of ICU patients (figure 2). Meta‑regression analysis (appendix p 11) showed that, compared with being in hospital generally, being in the ICU was associated with higher odds of secondary infections (adjusted OR 7·52 [95% CI 4·69–12·06]) and unspecified bacterial infections (4·47 [1·53–13·05]).

Microbiological data from 17 423 bacterial isolates were reported in 130 (88%) studies. The most common organisms detected were *S aureus* (n=2584 isolates [15%]), *Klebsiella* spp (n=2543 [15%]), and *Pseudomonas* spp (n=1830 [10%]). Distribution of organisms differed among patients with bacterial co-infections (n=1248) and those with secondary infections (n=6301). In co-infections, *S aureus* (n=258 [21%]), coagulase-negative staphylococci (n=171 [14%]), and *E coli* (n=153 [12%]) were the most common isolates, whereas in secondary infections, *Klebsiella* spp (n=993, [16%]), *Pseudomonas* spp (n=777 [12%]), and *S aureus* (n=753 [12%]) were most common (appendix p 12).

Reporting of antimicrobial resistance varied substantially between studies. In 93 (63%) studies, susceptibility or resistance data were reported for at least one species, and in 62 (42%) susceptibility or resistance data were reported for one or two species. Resistance or susceptibility data were most commonly reported for *S aureus* (n=73 studies), *Pseudomonas* spp (n=29), *Klebsiella* spp (n=28), and *Enterococcus* spp (n=27). Among studies in which antimicrobial resistance data were reported for at least four species (or for all reported species if fewer than four species were isolated), the prevalence of bacterial infections resistant to antimicrobials was 60·8% (95% CI 38·6–79·3; data from 17 studies), and the proportion of isolates that were antimicrobial resistant was 37·5% (26·9–49·5; data from 42 studies; appendix p 14). Sensitivity analyses of the prevalence of antimicrobial resistance stratified by quality score showed similar findings (appendix p 15). There was substantial heterogeneity in the reported prevalence of antimicrobial resistance: *I*^2^ was 85·8% (95% CI 78·8–90·6) for the per-patient analysis and 95·5% (94·5–96·2) for the per-organism analysis.

The highest prevalence of resistance was reported for multidrug-resistant *Stenotrophomonas* spp (100% [95% CI 82·6–100·0%]), A*cinetobacter* spp (96·5% [86·9–99·1] were multidrug resistant and 95·9% [84·1 –99·0] were resistant to carbapenems), and *Klebsiella* spp (88·3% [6·7–99·9] were resistant to colistin and 69·2% [49·6–83·6] to carbapenems; table 1). Meta-regression (adjusted for age and COVID-19 severity) showed that being in the ICU (adjusted OR 3·69 [95% CI 1·27–10·68]), receiving IL-6 inhibitors (1·44 [1·03–2·01]), and having diabetes (1·95 [1·19–3·21]) were associated with increased odds of the presence of antibiotic-resistant isolates (table 2).

**Table 1:** Antibiotic resistance in patients with COVID-19 and bacterial infections

		**Studies (n)**	Patients (n/N)*	**Prevalence (95% CI)**	**Heterogeneity *I*^2^ (95% CI)**
Meticillin-resistant *Staphylococcus aureus*		73	614/1529	41·1% (33·9–48·8)	52·4% (37·8–63·6)
Vancomycin-resistant *Enterococcus*		27	127/612	18·8% (13·3–25·8)	25·7% (0·0–53·8)
*Streptococcus pneumoniae*					
	Any resistance	2	3/7	58·4% (2·7–98·6)	0%
	Penicillin-resistant *S pneumoniae*	2	3/8	39·1% (5·0–88·7)	0% (0·0–89·6)
	Fluoroquinolone-resistant *S pneumoniae*	1	1/2	50·0% (5·9–94·1)	NA
*Pseudomonas* species					
	Any resistance	29	230/557	42·1% (25·0–61·2)	65·5% (49·0–76·7)
	Multidrug resistance	14	127/376	26·2% (10·7–51·1)	84·4% (75·3–90·2)
	Carbapenem resistance	16	53/182	29·2% (9·3–62·5)	9·1% (0·0–46·0)
	Produces ESBL or resistant to third-generation cephalosporins	5	49/66	59·9% (7·4–96·5)	0% (0·0–74·6)
	Colistin resistance	2	1/20	5·0% (0·7–28·2)	0%
*Klebsiella* species					
	Any resistance	28	911/1087	77·9% (53·2–91·6)	81·9% (74·6–87·0)
	Multidrug resistance	6	62/138	38·2% (36·4–40·0)	0% (0·0–74·6)
	Carbapenem resistance	20	701/902	69·2% (49·6–83·6)	85·6% (79·1–90·1)
	Produces ESBL or resistant to third-generation cephalosporins	16	411/603	47·4% (26·8–68·9)	91·6% (87·9–94·1)
	Colistin resistance	5	337/442	88·3% (6·7–99·9)	89·0% (77·1–94·7)
*Escherichia coli*					
	Any resistance	25	108/210	57·6% (38·0–75·0)	52·0% (24·1–69·6)
	Carbapenem resistance	5	23/93	18·6% (7·6–39·0)	0% (0·0–79·2)
	Produces ESBL or resistant to third-generation cephalosporins	11	27/126	21·1% (9·9–39·5)	4·5% (0·0–62·0)
	Multidrug resistance	22	111/233	52·1% (39·1–64·9)	35·0% (0·0–61·2)
	Colistin resistance	5	1/21	4·8% (0·7–27·1)	0% (0·0–89·6)
*Acinetobacter* species					
	Any resistance	20	791/890	95·0% (84·6–98·5)	85·7% (79·2–90·1)
	Multidrug resistance	16	551/583	96·5% (86·9–99·1)	59·3% (29·4–76·5)
	Carbapenem resistance	12	440/488	95·9% (84·1–99·0)	74·7% (55·3–85·7)
	Colistin resistance	6	242/398	37·7% (15·7–66·2)	96·1% (93·6–97·6)
*Enterobacter* species					
	Any resistance	9	34/53	64·0% (49·6–76·3)	0% (0·0–64·8)
	Multidrug resistance	3	12/25	48·0% (29·6%–66·9)	0·4% (0·0–89·6)
	Carbapenem resistance	6	10/27	37·0% (21·2–56·2)	0% (0·0–74·6)
	Produces ESBL or resistant to third-generation cephalosporins	6	15/29	51·7% (34·1–68·9)	0% (0·0–74·6)
Multidrug-resistant *Stenotrophomonas* species		6	10/14	100% (82·6–100·0)	0% (0·0–74·6)
*Serratia* species					
	Any resistance	3	1/8	12·5% (1·7–53·7)	0% (0·0–89·6)
	Produces ESBL or resistant to third-generation cephalosporins	1	1/3	33·3% (4·3–84·6)	NA
*Proteus* species					
	Any resistance	3	1/9	11·1% (1·5–50·0)	0% (0·0–89·6)
	Multidrug resistance	1	1/5	20·0% (2·7–69·1)	NA

Multidrug resistance was defined as non-susceptibility to at least one agent across three or more antibiotic classes (or as defined by study authors). NA=not applicable. ESBL=extended-spectrum β-lactamase.

* Proportion of patients infected with an antimicrobial-resistant organism among patients infected with any bacterial infection; studies reporting any susceptibility or resistance for that organism–drug combination contribute to the denominator.

**Table 2:** Predictors of antibiotic resistance in patients with bacterial infections and COVID-19

	**Per-patient resistance (n=17)**			**Per-organism resistance (n=42)**		
	Unadjusted odds ratio	Adjusted odds ratio	Studies	Unadjusted odds ratio	Adjusted odds ratio	Studies
Setting*						
Hospital inpatients	Ref	Ref	4	Ref	Ref	18
Intensive care unit	3·18 (0·23–43·85)	4·47 (0·36–55·70)	5	3·69 (1·28–10·63)	3·69 (1·27–10·68)	13
Hospital inpatients and outpatients	0·00 (0·00–not reached)	0·00 (0·00–not reached)	1	0·00 (0·00–not reached)	0·00 (0·00–not reached)	1
**Infection site**						
Respiratory system	Ref	Ref	3	Ref	Ref	4
Blood	0·12 (0·00–7·35)	0·02 (0·00–0·66)	1	0·15 (0·02–1·12)	0·06 (0·01–0·40)	7
Multiple	0·15 (0·01–2·21)	0·08 (0·01–0·70)	6	0·45 (0·08–2·64)	0·30 (0·06–1·51)	19
**Infection type**						
Both or unspecified	Ref	Ref	4	Ref	Ref	11
Co-infection	..	..	0	0·24 (0·01–5·78)	0·24 (0·01–5·37)	1
Secondary infection	4·89 (0·34–70·59)	1267·85 (0·14–not reached)	6	1·72 (0·53–5·54)	0·77 (0·18–3·25)	18
**Study end month**						
January–June, 2020	Ref	Ref	7	Ref	Ref	21
July–December, 2020	1·63 (0·47–5·58)	2·32 (0·76–7·10)	2	2·42 (0·56–10·53)	2·02 (0·46–8·89)	5
January–June, 2021	>1000 (0·00–not reached)	>1000 (0·00–not reached)	1	5·21 (1·01–26·96)	3·74 (0·72–19·53)	4
**Country income**						
High income	Ref	Ref	6	Ref	Ref	20
Low income or middle income	10·49 (1·03–106·97)	14·15 (1·85–108·33)	4	8·01 (3·19–20·09)	10·38 (3·97–27·00)	10
Multiple locations	..	(No data)	0	0·22 (0·03–1·88)	0·24 (0·03–1·94)	1
**WHO region**						
European	Ref	Ref	4	Ref	Ref	16
African	..	..	0	2·88 (0·39–21·07)	2·46 (0·34–17·68)	1
Eastern Mediterranean	>1000 (0·00–not reached)	>1000 (0·00–not reached)	1	25·60 (6·98–93·90)	27·13 (7·30–100·92)	2
Americas	0·00 (0·00–not reached)	0·00 (0·00–not reached)	1	1·23 (0·37–4·11)	2·07 (0·54–7·88)	4
South-East Asia	2·16 (0·86–5·42)	2·13 (0·78–5·83)	2	5·94 (1·30–27·17)	6·43 (1·38–29·95)	2
Western Pacific	6·34 (1·68–24·01)	5·62 (1·00–31·39)	2	8·82 (2·72–28·64)	6·85 (2·11–22·27)	4
Multiple	..	..	0	0·22 (0·03–1·88)	0·24 (0·03–1·94)	1
**Patient or treatment characteristics**†						
Age	5·76 (0·43–77·55)	12·51 (0·67–234·38)	10	1·01 (0·30–3·45)	1·29 (0·38–4·34)	31
Female sex	0·94 (0·26–3·39)	0·85 (0·19–3·91)	10	0·91 (0·50–1·66)	1·31 (0·65–2·64)	31
Mechanical ventilation*	1·19 (0·82–1·72)	1·30 (0·90–1·88)	8	1·16 (0·96–1·39)	1·17 (0·96–1·41)	25
Smoker	(Insufficient data)	(Insufficient data)	1	..	..	2
Obstructive pulmonary disease	0·09 (0·03–0·28)	(Insufficient data)	4	0·61 (0·14–2·71)	0·71 (0·17–2·94)	14
Cardiovascular disease	23·04 (0·40–1313·44)	118·78 (7·78–1814·67)	7	1·15 (0·41–3·25)	1·59 (0·60–4·27)	17
Diabetes	3·46 (1·61–7·46)	3·14 (2·19–4·50)	8	1·95 (1·23–3·11)	1·95 (1·19–3·21)	25
Malignancy	0·11 (0·02–0·79)	0·06 (0·02–0·18)	6	0·77 (0·57–1·03)	0·77 (0·56–1·04)	16
Immunocompromised	0·74 (0·12–4·65)	(Insufficient data)	4	0·71 (0·49–1·03)	0·71 (0·49–1·03)	13
Corticosteroids	1·26 (0·77–2·08)	1·65 (0·82–3·33)	8	1·28 (1·02–1·61)	1·33 (0·98–1·80)	22
Interleukin-6 inhibitor	1·49 (0·90–2·47)	1·70 (1·12–2·56)	6	1·36 (1·05–1·76)	1·44 (1·03–2·01)	17
Antibiotics	3·15 (0·98–10·10)	2·70 (1·28–5·70)	7	1·53 (0·84–2·78)	1·49 (0·82–2·71)	19

Adjusted data were adjusted for age and severity of COVID-19 infection. Ref=reference.

* Adjusted for age only.

† Odds ratios are for every 10% increase in the proportion of patients with comorbidities or receiving COVID-19 therapeutics, or for every 10-year increase in age.

Compared with the Americas region, the odds of antibiotic-resistant isolates were higher in studies done in the Eastern Mediterranean, South-East Asia, and Western Pacific regions (table 2). On both a per-patient and per-organism level, the odds of antimicrobial resistance were higher in studies done in low-income and middle-income countries than in those done in high-income countries (table 2). In per-patient adjusted analyses, cardiovascular disease (118·78 [95% CI 7·78–1814·67]), diabetes (3·14 [CI 2·19–4·50]), IL-6 inhibitor use (1·70 [CI 1·12–2·56]), and antibiotics use (2·70 [1·28–5·70]) were associated with increased odds of being infected with a resistant pathogen (table 2).

Meta-regression scatter plots show the association between continuous variables and odds of antimicrobial resistance (appendix pp 16–17). The appendix (p 13) also contains a summary of key data for the prevalence of bacterial infection and of antimicrobial resistance.

## Discussion

In this systematic revie w and meta-analysis of data from 148 studies and 362 976 patients, we identified a low prevalence of bacterial co-infection and a moderate prevalence of secondary infection in hospitalised patients with COVID-19. Patients hospitalised for COVID-19 in ICUs were at higher risk of bacterial infection than general hospitalised populations. In patients with COVID-19 and a bacterial co-infection, antimicrobial resistance is prevalent in approximately two-thirds of bacterial infections and approximately one-third of isolates.

Findings from this systematic review corroborate evidence from previous reviews^3^, ^5^, ^6^ that bacterial co-infection in patients with COVID-19 is low and that antibiotic therapy should not be given as standard unless bacterial infection is strongly suspected. In patients admitted to ICU, although co-infections are still infrequent, the risk of bacterial secondary infections is high, and therefore a careful risk–benefit assessment informed by clinical judgement, patient factors, and local epidemiology is advised before initiation of antibiotics.

WHO has identified a list of priority bacterial pathogens that are of public health importance and for which new and effective antibiotic treatments are urgently needed because of antimicrobial resistance.^13^ We found substantial prevalence of antimicrobial resistance for several WHO critical pathogens, including carbapenem-resistant *Acinetobacter baumannii* (96% of isolates were carbapenem resistant) and carbapenem-resistant Enterobacterales (69% of *Klebsiella spp* were carbapenem resistant). The high prevalence of resistance, combined with the high prevalence of these organisms in COVID-19 secondary infections, highlights the urgency of antimicrobial stewardship and the development of new therapeutic agents.

The odds of isolates being resistant to antimicrobials were ten times higher in studies done in low-income and middle-income countries than in those done in high-income countries. Low-income and middle-income countries face unique challenges including a high burden of infectious diseases and low access to—and capacity and resources for—diagnostics and surveillance.^168^ Our findings highlight the importance of continuous investments in building robust and sustainable systems to detect and monitor antimicrobial resistance and to strengthen capacity for antimicrobial stewardship and infection prevention and control in these settings. Antimicrobial resistance was more prevalent in studies of ICU populations than in those of general hospital populations. Patients in ICUs are more likely to require invasive interventions (eg, mechanical ventilation, intravascular access) than other hospitalised patients, predisposing them to nosocomial infection and antibiotic treatment, which in turn can select for resistant strains. This observation is consistent with pre-COVID-19 findings suggesting that ICU patients, and particularly those for whom invasive interventions are used, are at increased risk of hospital-acquired infections.^169^, ^170^ In our analysis, antibiotic exposure was a significant risk factor for antimicrobial resistance, consistent with mechanisms described in the literature.^171^, ^172^ IL-6 inhibitor use and diabetes were also associated with higher proportions of antimicrobial resistance at the patient level. These associations may be due to residual confounding, in that people with diabetes or who received IL-6 inhibitors might represent sub-populations with more severe COVID-19 who thus might stay in hospital for longer, leading to increased antibiotic use. Our study has several strengths, including its large sample size and substantial geographical coverage (148 studies from >40 countries). However, it also has some notable limitations. Many studies provided limited information about resistance or included resistance data for only one or two common resistant organisms. Therefore, it was challenging to generate a precise estimate of the true prevalence of antimicrobial resistance in people with COVID-19. We attempted to overcome this potential reporting bias by restricting our analyses to studies in which susceptibility or resistance data for four or more species were reported (or all species if fewer than four were reported). Despite these efforts, there is still a risk that we have underestimated the prevalence of clinically important antibiotic resistance, because we captured only the resistant organisms of greatest concern. Conversely, our results could potentially overestimate resistance if antimicrobial resistance is more likely to be reported in settings where it is more prevalent. Similarly, microbiological sampling might be more common in patients not responding to antimicrobial therapy, thereby potentially leading to overestimation of the prevalence of resistance.

Another key limitation was under-representation of regions outside North America and Europe, particularly low-income and middle-income countries. Our comparisons of resistance across regions thus had a high degree of heterogeneity and wide 95% CIs, suggesting the low precision of the estimates. Meta-regression, although useful for detecting variables with large associations with antimicrobial resistance, was probably underpowered to detect smaller associations given the small sample size of studies that comprehensively assessed antimicrobial resistance.

Our focus on antimicrobial resistance in patients with COVID-19 provides a granular look at prevalence and predictors for resistance in this population but does not address the societal risk of resistance in the broader context of COVID-19. Given the risk of transmission of antibiotic-resistant organisms beyond individual patient and health-care environments, an assessment of the downstream effects of resistance beyond hospitalised patients with COVID-19 will be important in future research. Preliminary data suggest that antimicrobial resistance could have increased during the pandemic, particularly for certain Gram-negative pathogens (eg, drug-resistant *Acinetobacter* spp, extended-spectrum β-lactamase-producing Enterobacterales). Additional large population-based studies with data for individual patients will provide more insight into the predictors of antimicrobial resistance.^173^, ^174^

In light of these limitations, our findings provide an impetus to further assess antimicrobial resistance in patients with COVID-19 and emphasise the need for detailed surveillance and opportunities to prevent resistance. Furthermore, our findings have particular relevance to attempts to inform clinical management policies and guidance, quantify the global burden of antimicrobial resistance,^1^ and to explorations of how the COVID-19 pandemic might affect these estimates.

## Data sharing

Data and analytic code are publicly available online.

## Declaration of interests

We declare no competing interests.

## Footnotes

* Non-susceptibility to at least one drug across three or more antibiotic classes, or as defined.
