# Supplementary material for: Antimicrobial resistance in patients with COVID-19: a systematic review and meta-analysis
Source: Lancet Microbe. 2023 Mar;4(3):e179–91. doi: 10.1016/S2666-5247(22)00355-X (PMC9889096; doi:10.1016/S2666-5247(22)00355-X)

THE LANCET
Microbe

Supplementary appendix

This appendix formed part of the original submission and has been peer reviewed. We post it as supplied by the authors.

Supplement to: Langford BJ, So M, Simeonova M, et al. Antimicrobial resistance in patients with COVID-19: a systematic review and meta-analysis. *Lancet Microbe* 2023; published online Jan 31. https://doi.org/10.1016/ S2666-5247(22)00355-X.

Supplementary Material

**Search Criteria**	**Page 2-3**
**Characteristics of Studies Reporting Bacterial Infections in COVID-19**	**Page 4-10**
**Predictors of Bacterial Infections in Patients with COVID-19**	**Page 11**
**Bacterial Pathogens in Patients with COVID-19**	**Page 12**
**Summary of Overall Prevalence of Bacterial Infection and Antibiotic Resistance in Patients with COVID-19**	**Page 13**
**Prevalence of Antibiotic Resistance in Patients with COVID-19**	**Page 14**
**Prevalence of Antibiotic Resistance in Patients with COVID-19 Stratified by Risk of Bias**	**Page 15**
**Scatterplots of Log Odds of Per-Patient Antimicrobial Resistance Based on Patient Characteristics**	**Page 16**
**Scatterplots of Odds of Per-Organism Antimicrobial Resistance Based on Patient Characteristics**	**Page 17**

Search Criteria

#		**Results** 01/12/2021
**1 - Coinfection**	Co-infect* OR coinfect* OR superinfect* OR "super infection"~3 OR "super infections"~3 OR "secondary infection"~3 OR "secondary infections"~3 OR "concomitant infection"~3 OR "concomitant infections"~3 OR "mixed infection"~3 OR "mixed infections"~3 OR co-exist* OR "cross infection"~3 OR "cross infections"~3 OR polymicrobial* OR "hospital acquired" OR "healthcare associated infection" OR "healthcare associated infections" OR ab:HAI OR ti:HAI OR nosocomial* OR "ventilator associated"~3 OR superimpose*	6,806
**2 - Bacterial**	*bacteri* OR "gram negative" OR "gram positive" OR "other pathogens"~3 OR antimicrobial* OR antibiotic* OR "microbial resistant" OR "microbial resistance" OR "drug resistant" OR "drug resistance" OR "culture positive" OR "culture negative" OR sepsis OR "blood infection"~3 OR "blood infections"~3 OR "bloodstream infection"~3 OR "bloodstream infections"~3 or pyemia OR pyaemia OR "skin disease" OR "skin diseases" OR "Lower respiratory tract infection" OR LRTI OR LRTIS OR septic*	17,963
**3 bacterial syndromes**	"bacterial pneumonia"~3 OR bacteriuria* OR bacteremia* OR bacteraemia* OR "urinary infection"~3 OR "urinary infections"~3 OR UTIs OR UTI OR pyelonephritis OR cystitis OR pyuria OR cellulitis OR "soft tissue infection" OR "soft tissue infections"	4,185
**#4**	(#1 AND #2) OR (#3)<br>((Co-infect* OR coinfect* OR superinfect* OR "super infection"~3 OR "super infections"~3 OR "secondary infection"~3 OR "secondary infections"~3 OR "concomitant infection"~3 OR "concomitant infections"~3 OR "mixed infection"~3 OR "mixed infections"~3 OR co-exist* OR "cross infection"~3 OR "cross infections"~3 OR polymicrobial* OR "hospital acquired" OR "healthcare associated infection" OR "healthcare associated infections" OR ab:HAI OR ti:HAI OR nosocomial* OR "ventilator associated"~3 OR superimpose*) AND (*bacteri* OR "gram negative" OR "gram positive" OR "other pathogens"~3 OR antimicrobial* OR antibiotic* OR "microbial resistant" OR "microbial resistance" OR "drug resistant" OR "drug resistance" OR "culture positive" OR "culture negative" OR sepsis OR "blood infection"~3 OR "blood infections"~3 OR "bloodstream infection"~3 OR "bloodstream infections"~3 or pyemia OR pyaemia OR "skin disease" OR "skin diseases" OR "Lower respiratory tract infection" OR LRTI	6,028

	OR LRTIS OR septic*)) OR ("bacterial pneumonia"~3 OR bacteriuria* OR bacteremia* OR bacteraemia* OR "urinary infection"~3 OR "urinary infections"~3 OR UTIs OR UTI OR pyelonephritis OR cystitis OR pyuria OR cellulitis OR "soft tissue infection" OR "soft tissue infections")	
**Part 1:**	#4 AND Entry_date:([20190101 TO 20210201])	2.816
**Part 2:**	#4 AND Entry_date:([20210201 TO 20211201])	3220

Supplementary Table 1. Characteristics of Studies Reporting Bacterial Infections in COVID-19 Patients

Author	Country	Setting	Age	Sample	Bacterial Co-Infection	Bacterial Secondary Infection	Bacterial infection Not Specified	Full AMR Evaluation$^{\Omega}$	Risk of Bias
Abelenda-Alonso, 2021	Spain	Hospital	64	2005		100 (5%)		Yes	low
AlSulaiman, 2021	Saudi Arabia	Hospital ICU	59	128		13 (10%)		No	moderate
Al-Karbolii MM, 2021	Iraq	Hospital		210	121 (58%)			No	low
Alser, 2021	United States	Hospital ICU	58	235			166 (71%)	No	moderate
Amer, 2021	Saudi Arabia	Hospital ICU	60	860			110 (13%)	No	moderate
Annane D, 2020	France	Hospital ICU	59	80		31 (39%)		No	moderate
Asmarawati, 2021	Indonesia	Hospital	52	218	10 (5%)	33 (15%)		No	low
Bardi T, 2021	Spain	Hospital ICU	61	140		57 (41%)		No	low
Bashir, 2021	Brunei	Hospital	35	180	8 (4%)			No	low
Baskaran, 2021	UK	Hospital ICU	59	254	14 (6%)	77 (30%)		No	low
Bhargava, 2021	United States	Hospital		290	22 (8%)	15 (5%)		Yes	moderate
Bhatt P, 2020	United States	Hospital	64	375		128 (34%)		Yes	low
Blonz, 2021	France	Hospital ICU	64	188	21 (11%)	92 (49%)		No	low
Bolker, 2021	United States	Hospital	60	228	29 (13%)	28 (12%)		No	low
Bonazzetti C, 2021	Italy	Hospital ICU	62	89		60 (67%)		Yes	moderate
Buetti, 2021	France	Hospital ICU	60	235		35 (15%)		No	moderate
Cataldo MA, 2020	Italy	Hospital ICU	62	57			23 (40%)	Yes	moderate
Catano-Correa, 2021	Colombia	Hospital		399		83 (21%)		No	low
Chauhdary W, 2020	Brunei	Hospital		141	5 (4%)			No	high
Chen S, 2021	China	Hospital	48	408	12 (3%)	10 (2%)		No	low
Cheng L, 2020	Hong Kong	Hospital	36	147	4 (3%)	8 (5%)		No	low
Chong W, 2021	United States	Hospital	63	244	8 (3%)	13 (5%)		No	low
Cohen R, 2021	Israel	Hospital	58	198	70 (35%)			Yes	low

Author	Country	Setting	Age	Sample	Bacterial Co-Infection	Bacterial Secondary Infection	Bacterial infection Not Specified	Full AMR Evaluation$^{\Omega}$	Risk of Bias
Cona A, 2021	Italy	Hospital	68	1351	18 (1%)	51 (4%)		Yes	low
Contou D, 2020	France	Hospital ICU	61	92	18 (20%)	8 (9%)		No	moderate
Copaja-Corzo C, 2021	Peru	Hospital ICU	54	124		50 (40%)		No	low
Cortes M, 2021	Brazil	Hospital ICU	58	73				No	moderate
COVID-ICU Group, 2021	France, Belgium, and Switzerland	Hospital ICU	63	4244	208 (5%)			No	low
D'Onofrio V, 2020	Belgium	Hospital	73	110	3 (3%)			No	low
DeVoe C, 2021	United States	Hospital	58	314		31 (10%)		No	moderate
Dolci A, 2020	Italy	Hospital	64	83			33 (40%)	No	high
Dravid A, 2021	India	Hospital	57	515		22 (4%)		Yes	moderate
Elabbadi A, 2021	France	Hospital	61	101	20 (20%)	48 (48%)		No	low
Esteban Ronda V, 2021	Spain	Hospital	63	208	20 (10%)			No	moderate
Evans T, 2021	UK	Hospital	67	130	0 (0%)			No	moderate
Falcone M, 2021	Italy	Hospital	69	315		69 (22%)		Yes	low
Fierens J, 2021	Belgium	Hospital ICU	62	74	5 (7%)	38 (51%)		No	moderate
Fontana C, 2021	Italy	Hospital	65	152			92 (61%)	Yes	moderate
Foschi C, 2021	Italy	Hospital ICU		178				No	
Garcia-Vidal C, 2021	Spain	Hospital	62	989	25 (3%)	38 (4%)		Yes	low
Gerver S, 2021	United Kingdom	Hospital/ Outpatient	55	223413	820 (<1%)	1098 (<1%)		No	low
Giacobbe D, 2020	Italy	Hospital ICU	66	78		31 (40%)		Yes	moderate
Giacobbe D, 2021	Italy	Hospital	66	165			5 (3%)	No	moderate
Goncalves Mendes Neto A, 2021	United States	Hospital	66	242			46 (19%)	No	high
Gragueb-Chatti I, 2021	France	Hospital ICU	64	151		98 (65%)		No	low
Grasselli G, 2021	Italy	Hospital ICU	62	774				Yes	moderate
Gudiol C, 2021	Multiple	Hospital	67	590		65 (11%)		Yes	moderate

Author	Country	Setting	Age	Sample	Bacterial Co-Infection	Bacterial Secondary Infection	Bacterial infection Not Specified	Full AMR Evaluation[Ω]	Risk of Bias
Gupta A, 2021	India	Hospital	41	89			37 (42%)	No	high
Ho KS, 2021	United States	Hospital	65	4313			265 (6%)	No	low
Hughes S, 2020	UK	Hospital	70	836			50 (6%)	No	moderate
Hughes S, 2021	UK	Hospital	65	594		45 (8%)	18 (3%)	No	moderate
Kaal A, 2021	Netherlands	Hospital	61	142	2 (1%)			No	low
Karaba SM, 2021	United States	Hospital	62	1016	43 (4%)			No	low
Karmen-Tuohy S, 2020	United States	Hospital	61	63		4 (6%)		No	moderate
Khatri A, 2021	United States	Hospital	63	13007		239 (2%)		No	low
Khurana S, 2021	India	Hospital		1179		151 (13%)		No	low
Kimmig L, 2020	United States	Hospital ICU	63	111		42 (38%)		No	moderate
Kokkoris S, 2021	Greece	Hospital ICU	64	50		20 (40%)		No	low
Kolenda C, 2020	France	Hospital ICU		99	15 (15%)			No	moderate
Kooistra E, 2021	Netherlands	Hospital ICU	65	189		71 (38%)		No	low
Kubin C, 2021	United States	Hospital	64	3028				Yes	low
Lardaro T, 2020	United States	Hospital	63	542	20 (4%)			No	low
Lehmann C, 2020	United States	Hospital	60	321	7 (2%)			No	low
Li J, 2020	China	Hospital		1479		102 (7%)		Yes	moderate
Llitjos J-F, 2021	France	Hospital ICU	63	176		92 (52%)		No	low
Luo X, 2021	China	Hospital	69	95				Yes	low
Luyt CE, 2020	France	Hospital ICU	48	50		43 (86%)		No	moderate
Lv Z, 2020	China	Hospital	62	354	18 (5%)			No	low
Mady A, 2020	Saudi Arabia	Hospital ICU	51	61		12 (20%)		No	low
Maes M, 2021	United Kingdom	Hospital ICU	62	81		39 (48%)		No	moderate
Mahmoudi, H, 2020	Iran	Hospital		340		43 (13%)		Yes	moderate
Martin A, 2021	United States	Hospital	69	198		24 (12%)		No	low
Martinez-Guerra BA, 2021	Mexico	Hospital	52	794	0 (0%)	54 (7%)		Yes	low

Author	Country	Setting	Age	Sample	Bacterial Co-Infection	Bacterial Secondary Infection	Bacterial infection Not Specified	Full AMR Evaluation$^{\Omega}$	Risk of Bias
May M, 2021	United States	Hospital/ Outpatient	65	2443	161 (7%)			No	low
Meawed T, 2021	Egypt	Hospital ICU	65	197		197 (100%)		Yes	low
Milas S, 2021	Belgium	Hospital	60	164	19 (12%)	9 (5%)		No	low
Ming DK, 2021	United Kingdom	Hospital	67	237			58 (24%)	No	moderate
Moolla MS, 2021	South Africa	Hospital ICU	53	363	20 (6%)	73 (20%)		No	low
Nasir N, 2021	Pakistan	Hospital	60	100				Yes	low
Nassar Y, 2021	Egypt	Hospital ICU	60	160			31 (19%)	No	moderate
Nebreda-Mayoral T, 2021	Spain	Hospital	73	712	39 (5%)	80 (11%)		Yes	moderate
Nori P, 2021	United States	Hospital		4267	152 (4%)			Yes	moderate
Nseir S, 2021	France, Ireland, Spain, Portugal, Denmark, Greece	Hospital ICU	64	568		205 (36%)		No	low
O'Kelly B, 2021	Ireland	Hospital	60	292			34 (12%)	No	moderate
Obata R, 2020	United States	Hospital	63	226		33 (15%)		No	low
Oliva A, 2020	Italy	Hospital ICU	67	55			40 (73%)	No	moderate
Ong C, 2021	Singapore	Hospital ICU	52	71		10 (14%)		No	moderate
Palanisamy N, 2021	India	Hospital ICU	62	750		64 (9%)		Yes	low
Pasquini Z, 2021	Italy	Hospital		1182			107 (9%)	No	low
Payson A, 2021	United States	Hospital	<1	53			4 (8%)	No	moderate
Perez-Garcia CN, 2021	Spain	Hospital	83	324			17 (5%)	No	moderate
Pettit N, 2021	United States	Hospital	66	148		15 (10%)		No	low
Petty L, 2021	United States	Hospital	65	2205	66 (3%)	75 (3%)		No	low
Pickens C, 2020	United States	Hospital ICU	62	179	28 (16%)	72 (40%)		No	low
Pink I, 2020	Germany	Hospital	57	99			32 (32%)	no	low
Posteraro B, 2021 (1)	Italy	Hospital ICU		150			97 (65%)	No	moderate

Author	Country	Setting	Age	Sample	Bacterial Co-Infection	Bacterial Secondary Infection	Bacterial infection Not Specified	Full AMR Evaluation$^{\Omega}$	Risk of Bias
Posteraro B, 2021 (2)	Italy	Hospital		293			44 (15%)	Yes	low
Putot A, 2020	France and Switzerland	Hospital	86	914		72 (8%)		No	low
Ramadan HKA, 2020	Egypt	Hospital		260	28 (11%)			Yes	moderate
Ramos R, 2021	Spain	Hospital ICU	61	213		95 (45%)		Yes	low
Rajni E, 2021	India	Hospital	52	158			14 (9%)	No	moderate
Rakiro J, 2021	Kenya	Hospital ICU	61	321	5 (2%)	62 (19%)		Yes	low
Rana MA, 2021	Pakistan	Hospital ICU		400		90 (23%)		No	
Razazi K, 2020	France	Hospital ICU	59	90	14 (16%)	56 (62%)		No	low
Ripa M, 2020	Italy	Hospital	64	731		68 (9%)		Yes	low
Risa E, 2021	United States	Hospital ICU	59	126		77 (61%)		No	low
Ritter LA, 2021	United States	Hospital ICU	52	135		85 (63%)		No	low
Rothe K, 2020	Germany	Hospital	64	140	8 (6%)		38 (27%)	Yes	moderate
Rouyer M, 2021	Germany	Hospital ICU	62	79		42 (53%)		No	low
Rouze A, 2021 (1)	Europe (France, Spain, Greece, Portugal, Ireland)	Hospital ICU	64	568	55 (10%)			No	moderate
Rouze A, 2021 (2)	Europe (France, Spain, Greece, Portugal, Ireland)	Hospital ICU	64	568		287 (51%)		No	low
Ruiz-Bastian M, 2021	Spain	Hospital		1195		66 (6%)		Yes	moderate
Saade A, 2021	France	Hospital ICU	59	100	7 (7%)	29 (29%)		No	low
Saeed N, 2021	Bahrain	hospital	50	1380	98 (7%)	185 (13%)		Yes	low
Salamat MS, 2021	Phillipines	Hospital	56	200	22 (11%)			no	low
Sang L, 2021	China	Hospital ICU	63	190				Yes	low
Sarikaya B, 2020	Turkey	Hospital	61	179		25 (14%)		No	moderate
Scott H, 2021	United States	Hospital	61	1389	28 (2%)	88 (6%)		No	low
Senok A, 2021	United Arab Emirates	Hospital		29802			392 (1%)	No	moderate

Author	Country	Setting	Age	Sample	Bacterial Co-Infection	Bacterial Secondary Infection	Bacterial infection Not Specified	Full AMR Evaluation$^{\Omega}$	Risk of Bias
Sepulveda J, 2020	United States	Hospital		4478	170 (4%)			No	low
Shafran N, 2021	Israel	Hospital	68	642			81 (13%)	No	moderate
Sharma B, 2021	India	Hospital		1844	38 (2%)	108 (6%)		yes	moderate
Sharov KS, 2020	Russia	Hospital/Outpatient		1351	194 (14%)	300 (22%)		No	
Shukla B, 2021	United States	Hospital	69	918		50 (5%)		No	moderate
Signorini L, 2021	Italy	Hospital ICU	62	92		53 (58%)		No	low
Silva IC, 2021	Venezuela	Hospital	64	145	11 (8%)			No	moderate
Smith L, 2021	United States	Hospital	62	963		49 (5%)		No	low
Somers E, 2020	United States	Hospital ICU	58	154		62 (40%)		No	low
Son H-J, 2021	South Korea	Hospital	73	152			17 (11%)	Yes	low
Stevens R, 2021 (1)	United States	Hospital	64	654			49 (7%)	No	low
Stevens R, 2021 (2)	United States	Hospital/ Outpatient	45	346			1(<1%)	Yes	moderate
Suarez-de-la-Rica, 2021	Spain	Hospital ICU	62	107		46 (43%)		Yes	moderate
Sulaiman I, 2021	United States	Hospital ICU	65	589				No	moderate
Tang H, 2021	China	Hospital	49	142		21 (15%)		No	moderate
Tayyab N, 2021	Pakistan	Hospital ICU	58	114				Yes	moderate
Temperoni C, 2021	Italy	Hospital ICU	67	89				Yes	moderate
Thelen JM, 2021	Netherlands	Hospital/ Outpatient	70	678	7 (1%)			No	low
Townsend L, 2020	Ireland	Hospital	66	117	95 (81%)			No	low
Vanhomwegen C, 2021	Belgium	Hospital ICU	61	66	7 (11%)			No	low
Vaughn VM, 2021	United States	Hospital	65	1705	59 (3%)			No	low
Vijay S, 2021	India	Hospital		17534			640 (4%)	Yes	moderate
Wang L, 2021	United Kingdom	Hospital	67	1396	37 (3%)			No	low
Yang X, 2020	China	Hospital ICU	60	52		4 (8%)		Yes	moderate
Yarden Bilavski H, 2021	Israel	Hospital	<1	75		8 (11%)		No	low

Author	Country	Setting	Age	Sample	Bacterial Co-Infection	Bacterial Secondary Infection	Bacterial infection Not Specified	Full AMR Evaluation[Ω]	Risk of Bias
Yoke LH, 2021	United States	Hospital/ Outpatient	61	71	3 (4%)			No	low
Yu D, 2020	Sweden	Hospital	64	2240				Yes	moderate
Zhang G, 2020	China	Hospital	55	221			17 (8%)	No	moderate
Zhou A, 2021	China	Hospital	43	683		38 (6%)		No	low

Ω Full AMR evaluation indicates whether the study reported susceptibility and/or resistance for four or more species, or reported susceptibility/resistance for all reported species if fewer than four species were isolated.

Supplementary Table 2. Predictors of Bacterial Infections in Patients with COVID-19

Characteristic	Co-infection (n = 55)			Secondary infection (n = 81)			Unspecified bacterial infection (n = 30)		
	Unadjusted	Adjusted	Studies	Unadjusted	Adjusted	Studies	Unadjusted	Adjusted	Studies
**Setting**			46			72			25
Hospital	Reference	Reference	30	Reference	Reference	34	Reference	Reference	19
Hospital ICU	1.76 (0.78 to 4.00)	1.74 (0.77 to 3.92)	12	7.55 (4.70 to 12.11)	7.52 (4.69 to 12.06)	37	4.46 (1.53 to 13.03)	4.47 (1.53 to 13.05)	5
Hospital/Outpatient	0.35 (0.10 to 1.28)	0.33 (0.09 to 1.21)	4	0.06 (0.01 to 0.40)	0.06 (0.01 to 0.40)	1	0.01 (0.00 to 0.29)	0.01 (0.00 to 0.29)	1
**Body site**			41			68			22
Respiratory	Reference	Reference	9	Reference	Reference	14	Reference	Reference	5
Blood	0.10 (0.01 to 1.02)	0.13 (0.01 to 1.31)	1	0.21 (0.07 to 0.60)	0.26 (0.12 to 0.56)	12	1.47 (0.21 to 10.13)	1.00 (0.22 to 4.49)	3
Multiple	0.34 (0.15 to 0.80)	0.39 (0.17 to 0.90)	31	0.27 (0.12 to 0.64)	0.43 (0.24 to 0.79)	42	2.49 (0.61 to 10.08)	1.29 (0.39 to 4.32)	13
**Study end month**			41			68			22
Jan-Jun 2020	Reference	Reference	31	Reference	Reference	45	Reference	Reference	13
Jul-Dec 2020	0.82 (0.29 to 2.31)	0.91 (0.34 to 2.43)	7	0.52 (0.23 to 1.14)	0.69 (0.39 to 1.23)	17	0.97 (0.25 to 3.84)	0.55 (0.18 to 1.75)	6
Jan-Jun 2021	1.33 (0.30 to 5.95)	1.61 (0.36 to 7.25)	3	2.85 (0.83 to 9.83)	1.86 (0.72 to 4.77)	6	1.16 (0.20 to 6.79)	0.79 (0.18 to 3.47)	3
**WHO region**			41			68			22
Europe	Reference	Reference	22	Reference	Reference	33	Reference	Reference	11
Africa	0.37 (0.06 to 2.11)	0.26 (0.04 to 1.49)	2	0.59 (0.09 to 3.84)	0.32 (0.08 to 1.30)	2	(No data)	(No data)	0
Eastern Mediterranean	(No data)	(No data)	0	3.50 (0.69 to 17.86)	1.66 (0.49 to 5.65)	3	0.58 (0.08 to 4.24)	0.08 (0.02 to 0.38)	2
Americas	0.44 (0.19 to 1.02)	0.62 (0.26 to 1.49)	12	0.41 (0.19 to 0.85)	0.70 (0.40 to 1.24)	20	0.35 (0.09 to 1.45)	0.49 (0.18 to 1.31)	5
South-East Asia	0.57 (0.05 to 6.29)	1.27 (0.10 to 16.07)	1	0.22 (0.05 to 1.03)	0.30 (0.09 to 0.97)	3	0.81 (0.11 to 6.05)	2.38 (0.36 to 15.79)	2
Western Pacific	0.56 (0.16 to 2.03)	1.74 (0.20 to 15.16	4	0.18 (0.05 to 0.57)	0.40 (0.15 to 1.06)	6	0.31 (0.04 to 2.32)	0.61 (0.15 to 2.41)	2
Multiple	(No data)	(No data)	0	0.30 (0.02 to 4.04)	0.93 (0.13 to 6.57)	1	(No data)	(No data)	0
Age (10-year increase)	1.20 (0.70 to 2.06)	1.12 (0.67 to 1.88)	41	1.10 (0.77 to 1.58)	0.97 (0.75 to 1.25)	68	1.30 (0.58 to 2.90)	1.33 (0.69 to 2.58)	22
% Female (10%↑)	0.64 (0.43 to 0.97)	0.81 (0.43 to 1.51)	40	0.56 (0.42 to 0.76)	1.10 (0.80 to 1.51)	67	0.46 (0.27 to 0.76)	0.63 (0.35 to 1.11)	22
% Mech vent. (10%↑)	1.17 (1.04 to 1.32)	1.17 (1.04 to 1.32)	26	1.32 (1.23 to 1.42)	1.32 (1.23 to 1.42)	56	1.19 (1.00 to 1.42)	1.18 (1.00 to 1.40)	14
% Smoker (10%↑)	0.85 (0.48 to 1.50)	1.08 (0.65 to 1.79)	12	0.97 (0.68 to 1.38)	0.95 (0.75 to 1.20)	16	0.99 (0.12 to 8.44)	(Insufficient data)	4
% COPD (10%↑)	0.90 (0.48 to 1.70)	1.20 (0.77 to 1.87)	23	1.84 (0.85 to 3.99)	1.75 (1.00 to 3.07)	37	4.53 (1.10 to 18.70)	2.26 (0.46 to 11.25)	9
% CVD (10% ↑)	0.96 (0.73 to 1.27)	1.04 (0.80 to 1.36)	25	0.91 (0.64 to 1.30)	1.00 (0.75 to 1.35)	43	1.02 (0.43 to 2.43)	0.89 (0.44 to 1.80)	12
% Diabetes (10%↑)	1.18 (0.91 to 1.52)	0.93 (0.70 to 1.24)	32	1.54 (1.15 to 2.07)	1.07 (0.83 to 1.40)	54	1.30 (0.77 to 2.20)	0.70 (0.42 to 1.16)	14
% Malignancy (10%↑)	0.97 (0.78 to 1.21)	1.02 (0.85 to 1.23)	20	1.02 (0.79 to 1.32)	1.06 (0.88 to 1.28)	37	1.15 (0.81 to 1.63)	1.47 (1.12 to 1.93)	11
% Immunocomp. (10%↑)	1.28 (0.66 to 2.49)	1.41 (0.81 to 2.45)	22	0.80 (0.57 to 1.12)	0.97 (0.75 to 1.26)	36	1.33 (0.92 to 1.92)	1.63 (1.16 to 2.28)	12
% Corticosteroid (10%↑)	0.85 (0.67 to 1.08)	0.81 (0.67 to 0.98)	16	1.09 (0.95 to 1.24)	0.97 (0.88 to 1.07)	49	0.93 (0.72 to 1.22)	0.71 (0.62 to 0.82)	10
% IL-6 inhibitor (10%↑)	0.73 (0.55 to 0.96)	0.68 (0.56 to 0.83)	11	1.08 (0.92 to 1.27)	0.96 (0.84 to 1.10)	38	0.99 (0.72 to 1.36)	0.76 (0.57 to 1.00)	8

Adjustment variables included age and severity of COVID-19 infection. Setting and mechanical ventilation were only adjusted for age.
Mech vent: mechanical ventilation

Supplementary Table 3. Bacterial Pathogens in Patients with COVID-19

	Co-infection		Secondary infection		Both/ Unspecified		Total	
	**n**	**%**	**n**	**%**	**n**	**%**	**n**	**%**
**Gram-Positive**								
Coagulase-negative Staph.	171	14%	482	8%	352	4%	1005	6%
Enterococcus spp.	36	3%	668	11%	852	9%	1556	9%
Staphylococcus aureus	258	21%	753	12%	1573	16%	2584	15%
Streptococcus pneumoniae	65	5%	66	1%	278	3%	409	2%
Other *Streptococcus* spp.	28	2%	96	2%	364	4%	488	3%
Other Gram-positive	58	5%	129	2%	81	1%	268	2%
**Gram-Negative**								
Acinetobacter baumannii	25	2%	594	9%	713	7%	1332	8%
Citrobacter spp.	5	0%	53	1%	40	0%	98	1%
Escherichia coli	153	12%	414	7%	1100	11%	1667	10%
Haemophilus spp.	54	4%	26	0%	309	3%	389	2%
Enterobacter spp.	42	3%	194	3%	293	3%	529	3%
Klebsiella spp.	110	9%	993	16%	1440	15%	2543	15%
Moraxella spp.	14	1%	2	0%	13	0%	29	0%
Proteus spp.	24	2%	48	1%	44	0%	116	1%
Pseudomonas spp.	72	6%	777	12%	981	10%	1830	10%
Serratia spp.	5	0%	102	2%	186	2%	293	2%
Stenotrophomonas spp.	4	0%	200	3%	199	2%	403	2%
Other Gram-negative	17	1%	460	7%	691	7%	1168	7%
Other not reported	107	9%	244	4%	365	4%	716	4%

Supplementary Table 4. Summary of Overall Prevalence of Bacterial Infection and Antibiotic Resistance in Patients with COVID-19*

	Combined Hospitalized/ Community<br>% (95% CI)	Hospitalized Only<br>% (95% CI)	Intensive-Care Unit Only<br>% (95% CI)
**Prevalence of Bacterial Infection**			
Co-infection	2.7% (0.8 to 8.8%)	4.9% (3.2 to 7.6%)	8.4% (6.0 to 11.7%)
Secondary infection	3.6% (0.2 to 38.4%)	8.4% (6.7 to 10.3%)	39.9% (31.1 to 49.5%)
Both/unspecified	0.3% (0.0 to 2.0%)	11.2% (7.3 to 16.7%)	44.5% (24.2 to 66.8%)
**Prevalence of Antibiotic Resistance***			
Co-infection	(no data)	69.5% (2.2 to 99.6%)	(no data)
Secondary infection	(no data)	25.1% (12.5 to 43.9%)	54.5% (27.9% to 78.7%)
Both/unspecified	0.0% (0.0 to 100.0%)	33.8% (23.6% to 45.8%)	43.1% (36.3 to 50.1%)

*AMR data is denominated by organism (per-organism analysis)

Supplementary Figure 1. Prevalence of Antibiotic Resistance in Patients with COVID-19

Per-Patient

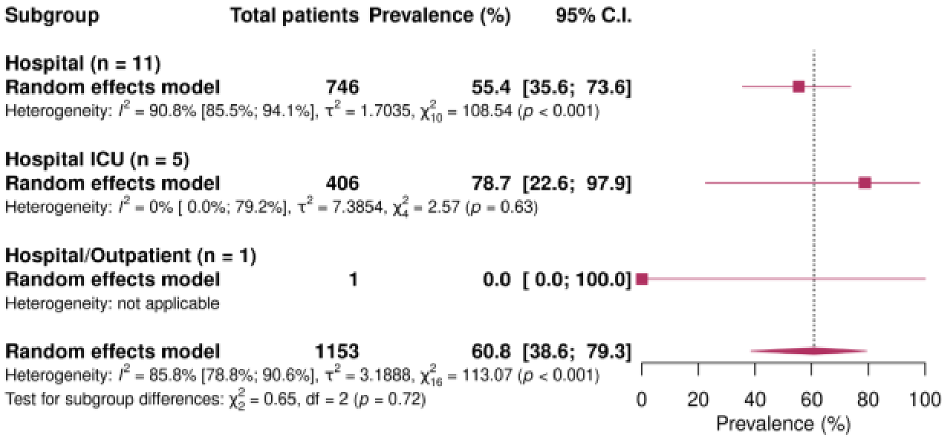

Per-Organism

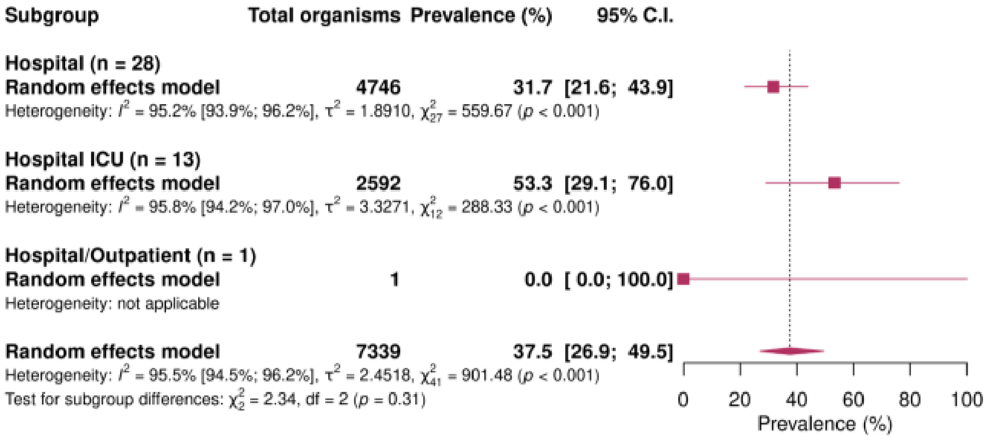

Supplementary Figure 2. Prevalence of Antibiotic Resistance in Patients with COVID-19 Stratified by Risk of Bias

Per-Patient

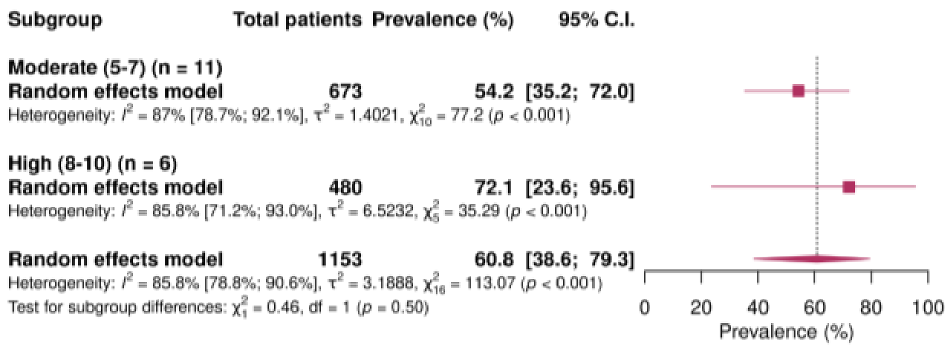

Per-Organism

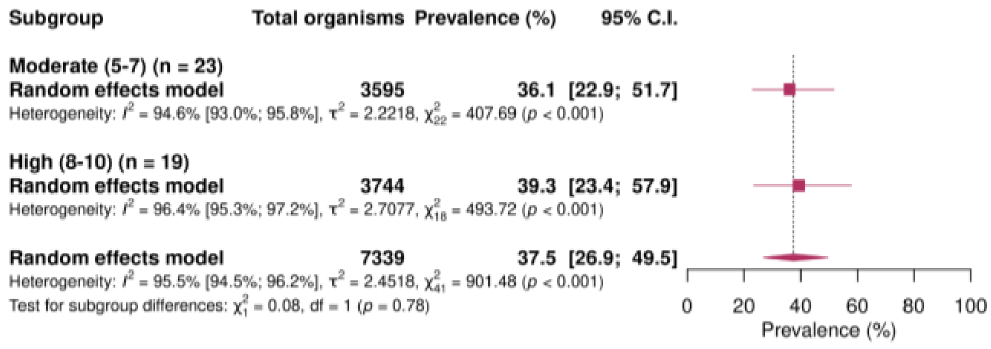

Supplementary Figure 3a. Scatterplots of Log Odds of Per-Patient Antimicrobial Resistance Based on Patient Characteristics

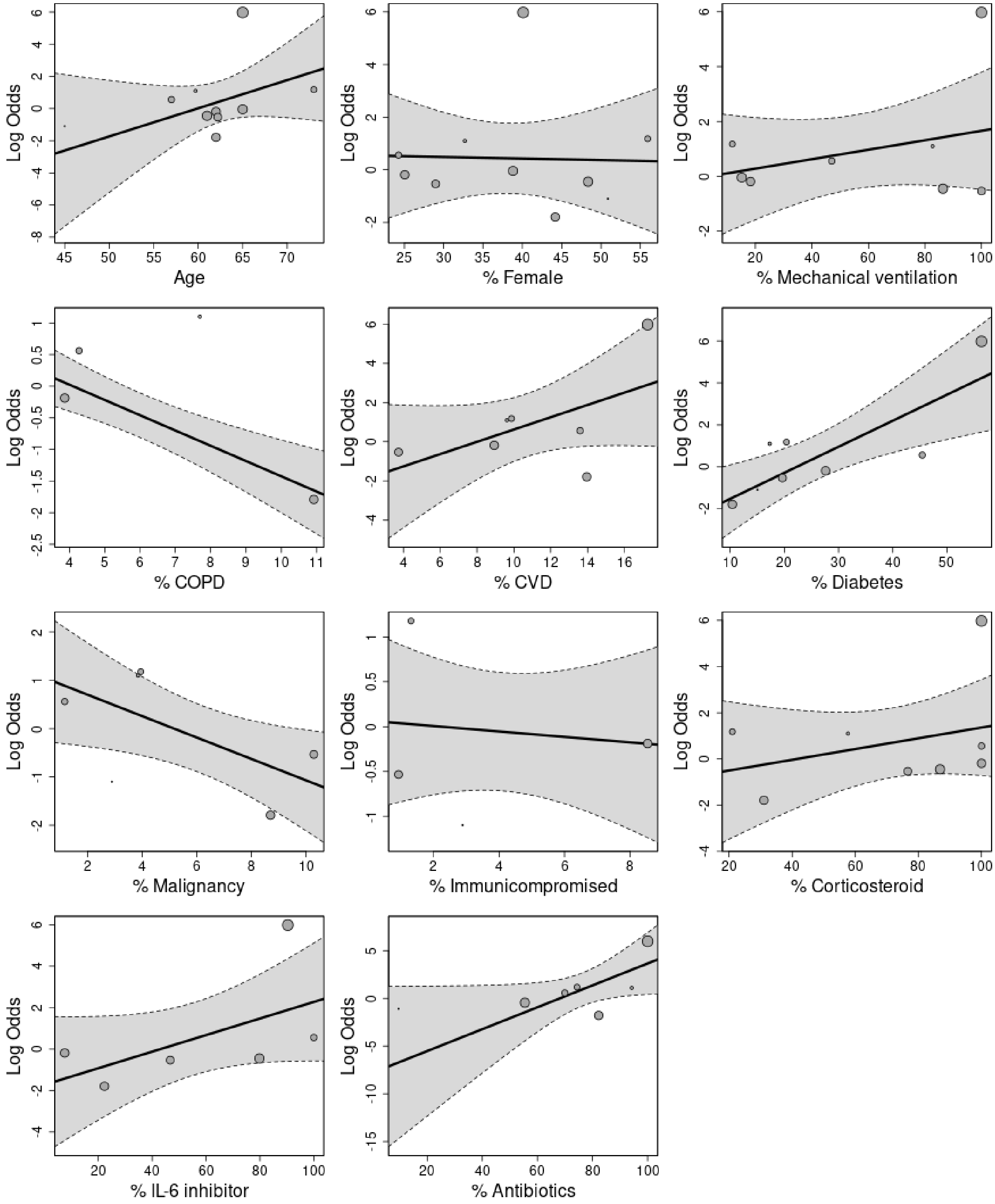

Supplementary Figure 3b. Scatterplots of Odds of Per-Organism Antimicrobial Resistance Based on Patient Characteristics

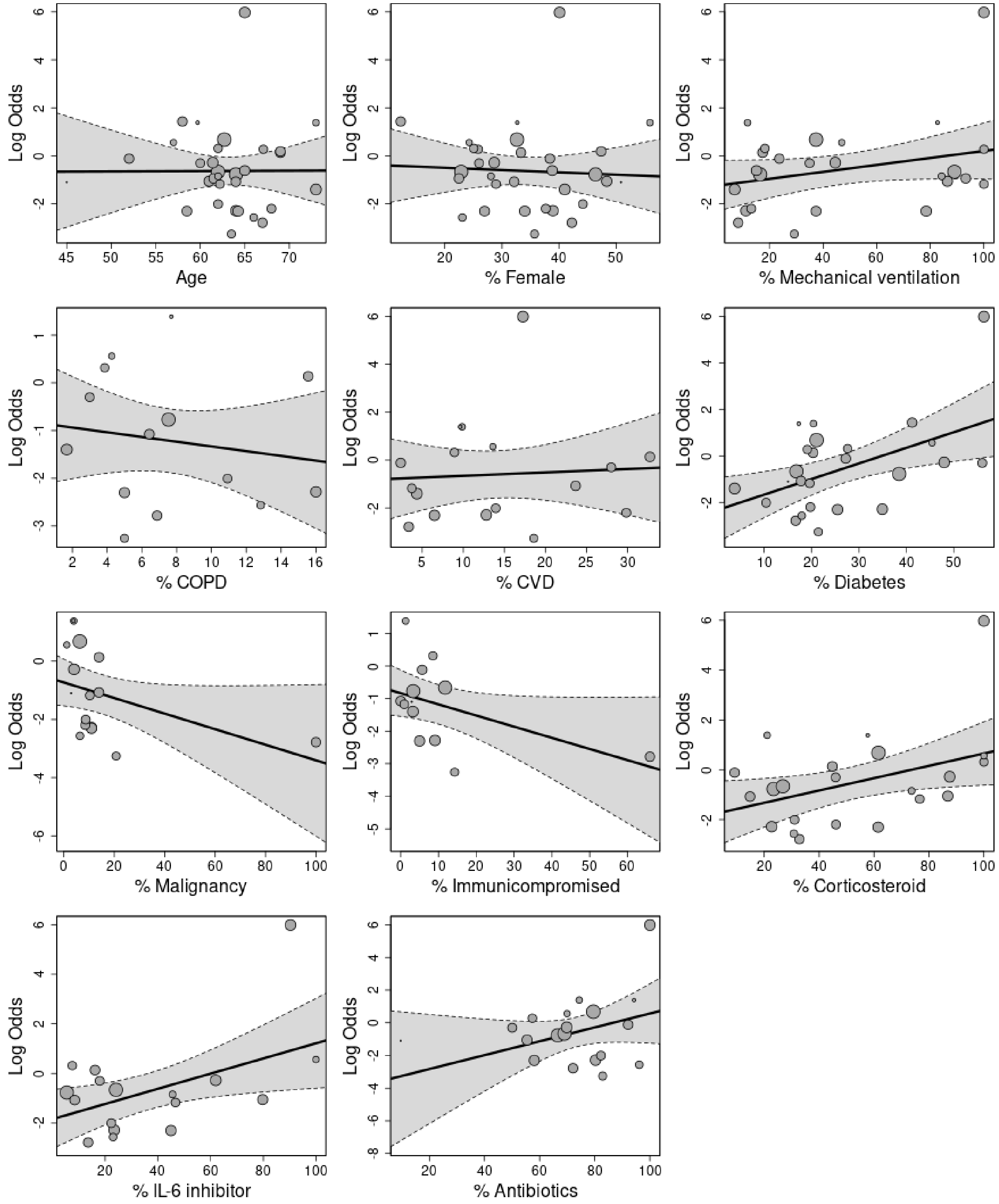

Supplement: Supplementary appendix [file mmc1.pdf]
